# Differential Gene Expression from Microarray Analysis Distinguishes Woven and Lamellar Bone Formation in the Rat Ulna following Mechanical Loading

**DOI:** 10.1371/journal.pone.0029328

**Published:** 2011-12-22

**Authors:** Jennifer A. McKenzie, Elise C. Bixby, Matthew J. Silva

**Affiliations:** 1 Department of Orthopaedics, Washington University, St. Louis, Missouri, United States of America; 2 Department of Biomedical Engineering, Washington University, St. Louis, Missouri, United States of America; University of Western Ontario, Canada

## Abstract

Formation of woven and lamellar bone in the adult skeleton can be induced through mechanical loading. Although much is known about the morphological appearance and structural properties of the newly formed bone, the molecular responses to loading are still not well understood. The objective of our study was to use a microarray to distinguish the molecular responses between woven and lamellar bone formation induced through mechanical loading. Rat forelimb loading was completed in a single bout to induce the formation of woven bone (WBF loading) or lamellar bone (LBF loading). A set of normal (non-loaded) rats were used as controls. Microarrays were performed at three timepoints after loading: 1 hr, 1 day and 3 days. Confirmation of microarray results was done for a select group of genes using quantitative real-time PCR (qRT-PCR). The micorarray identified numerous genes and pathways that were differentially regulated for woven, but not lamellar bone formation. Few changes in gene expression were evident comparing lamellar bone formation to normal controls. A total of 395 genes were differentially expressed between formation of woven and lamellar bone 1 hr after loading, while 5883 and 5974 genes were differentially expressed on days 1 and 3, respectively. Results suggest that not only are the levels of expression different for each type of bone formation, but that distinct pathways are activated only for woven bone formation. A strong early inflammatory response preceded an increase in angiogenic and osteogenic gene expression for woven bone formation. Furthermore, at later timepoints there was evidence of bone resorption after WBF loading. In summary, the vast coverage of the microarray offers a comprehensive characterization of the early differences in expression between woven and lamellar bone formation.

## Introduction

Osteoblasts produce either woven or lamellar bone tissue. Woven bone is characterized by its rapid deposition, random collagen arrangement and low density. By contrast, lamellar bone deposits more slowly, has well aligned collagen and higher density. Although many histological, structural and mechanical aspects that distinguish woven and lamellar bone have been described, the molecular mechanisms that distinguish these two dramatically different bone formation processes are not well understood. A better understanding of these molecular events could be the basis for future developments of novel therapeutics to promote either rapid or sustained bone formation.

Mechanical loading can induce formation of woven or lamellar bone in a controlled manner in the adult skeleton [1]. In particular, rat forelimb loading can be performed in a single bout to induce the formation of woven bone (WBF loading) [2]–[4], or formation of lamellar bone (LBF loading) [5]. WBF loading in the rat ulna stimulated early increases in cell proliferation and angiogenesis prior to woven bone formation [5]–[8]. By contrast, LBF loading did not increase cell proliferation, vessel volume or expression of angiogenic genes at early timepoints prior to lamellar bone formation [5]. However, these prior studies only examined select target genes. Thus, further work is required to broaden our understanding of the overall expression differences that distinguish woven and lamellar bone formation.

A microarray allows simultaneous measurement of the expression levels of thousands of genes [9], whereas more traditional analysis of individual genes using polymerase chain reaction (PCR) is impractical for analysis of a large number of coordinately expressed genes. Whole genome analysis has been used in studies of bone loading [10]–[12], fracture [13]–[16], and intramembranous bone regeneration [17]. However, prior studies have not clearly separated endocortical from periosteal bone formation, single-day from multiple-day loading regimes, or woven from lamellar bone formation.

The objective of our study was to identify gene expression differences between woven and lamellar bone formation. We induced woven and lamellar bone formation using a single bout of mechanical loading in the rat forelimb. WBF loading produces an ulnar stress fracture and subsequent healing through the formation of woven bone [2], [18]. LBF loading does not damage the ulna and induces the formation of lamellar bone [5]. Our focus was on three early timepoints after loading: 1 hr, 1 day and 3 days. A whole genome microarray, followed by validation of select results through qRT-PCR, was used to describe the gene expression profiles that differ between woven and lamellar bone formation.

## Methods

### Ethics Statement

The research described in this study involving animals is in compliance with all applicable Federal regulations and University and sponsoring agency policies and procedures. The study was approved by the Washington University Animal Studies Committee (protocol number 20090252).

### Forelimb Loading

**Figure 1:**
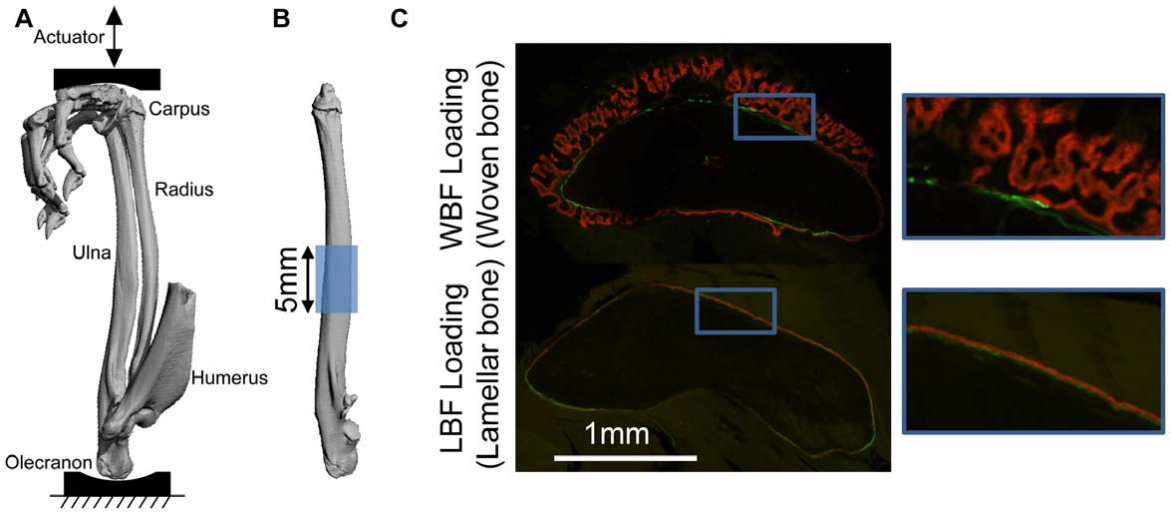
Mechanical loading was applied to the rat forelimb and a central region of the ulna was analyzed. (A) Medial view of bones in a right forelimb of a rat obtained by microCT during simulated loading (Reprinted from Journal of Biomechanics, 40, Uthgenannt BA & Silva MJ, 317–324, 2007, with permission from Elsevier). (B) The central 5 mm of the ulna and surrounding periosteum were isolated for microarray analysis. (C) Representative transverse histological sections from a previous study [5] that illustrate bone formation after loading. WBF loading leads to woven bone formation while LBF loading increases lamellar bone formation. After loading, fluorochrome labels were injected *in vivo* on days 3 (green) and 8 (red) prior to animal sacrifice on day 10. Plastic embedded transverse sections were taken 1 mm distal to the ulna midpoint.

The right forelimbs of adult male rats (Fischer F344, 5 mo., 337±24 g; Harlan) were loaded in axial compression using one of two loading protocols shown previously to active woven or lamellar bone formation [2], [5] (Table 1). Rats were anesthetized (1–3% isoflorane) prior to loading. The forelimb was positioned between two loading fixtures on a servohydraulic testing machine (Instron 1331) and a 0.3 N compressive preload was applied (Figure 1A). Two distinct loading protocols were used to stimulate formation of woven or lamellar bone at the ulnar mid-shaft. The first loading protocol, WBF loading, is a fatigue protocol that been shown to reduce ulnar strength and stiffness by over 50% [18] and induce abundant woven bone formation within 7 days [2]. Woven bone forms where loading-induced surface strains are highest (at the midshaft of the ulna), while increased lamellar bone forms at sites with lower strain levels. WBF loading applies a cyclic, haversine (2 Hz) waveform to the right forelimbs in a single bout (18 N peak force) until a prescribed increase in displacement (1.3 mm, 65% of fracture). The second loading protocol, LBF loading, does not reduce ulnar strength or stiffness after loading, and induces a significant increase in lamellar bone formation with no woven bone; for example, lamellar bone formation rate (BFR/BS) is increased by 80% at the ulnar mid-shaft in loaded limbs vs. controls [5]. LBF loading applies a trapezoidal waveform to the right forelimb in a single bout (0.5 s triangle load-unload to 15 N, followed by 9.5 s rest; 100 cycles). Both WBF and LBF loading waveforms have a load/unload period of 0.5 s per cycle. Following loading, all rats received analgesia (i.m. 0.05 mg/kg buprenorphine) and were allowed normal cage activity and ad libitum access to food and water.

**Table 1:** Loading parameter summary for the 48 rats used in the study.

	Non loaded	Num. of rats loaded			Applied force (N)	Loading cycles	Increase in disp. (mm)
		1 hr	Day 1	Day 3			
Woven		7	7	7	18	7501±5676	1.30
Lamellar		7	7	7	15	100	0.10±0.06
Normal	6				-	-	-

### Experimental Overview

**Figure 2:**
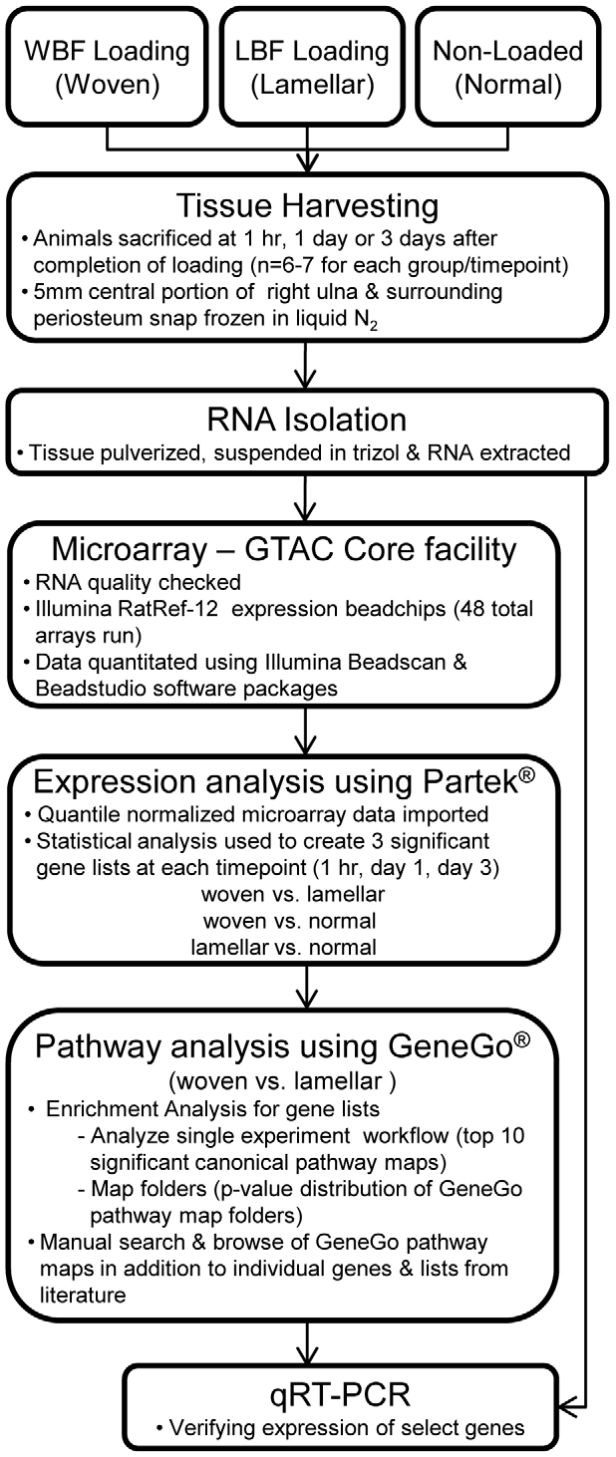
Flow chart describing the experiment and analysis. Three separate groups of animals were used to provide samples for WBF, LBF and Non-Loaded groups. Thus, comparisons between loading conditions are between (not within) animals.

The basic steps in experimental design and analysis are given in Figure 2. A total of 42 rats were euthanized at 1 hr, 1 or 3 days after the end of loading, corresponding to timepoints that were previously investigated [5], [7] and ulnae were dissected without delay. An additional six rats were not loaded and served as age-matched controls, referred to as ‘normal’ rats (Table 1). The right ulna (with only surrounding periosteal tissue) was frozen in liquid nitrogen within 5 minutes of animal death. Dissected bones were stored at −80 C until they were processed for RNA extraction.

### RNA Extraction and Preparation

Two transverse cuts were made to isolate the central 5 mm of the ulna and surrounding periosteum (Figure 1B), which was then pulverized and suspended in TRIzol (Invitrogen) as previously described [7]. This sample contains a mix of cell types from several tissues, including the original cortical bone, periosteal and sub-periosteal tissues where the nascent bone will form, and a small amount of bone marrow (although the marrow cavity is negligible at the ulnar mid-diaphysis in the F344 male rat). We did not attempt to isolate only the newly forming bone, as it is not yet present at the early timepoints we examined. Such an approach has been used by us [5], [7], [19], [20] and others [8], [11], [12] in previous studies of gene expression after mechanical loading. Total RNA was isolated using an RNeasy mini kit (Qiagen) and quantified (Nanodrop, ND-1000). RNA from each rat (48 rats total) was prepared for microarray analysis by the Washington University Genome Technology Access Center (Illumina, RatRef-12; 48 arrays total). The total RNA quality was determined by Agilent 2100 bioanalyzer (Agilent Technologies) according to manufacturer's recommendations. At the Genome Center, RNA transcripts were amplified by T7 linear amplification (MessageAmp TotalPrep amplification kit; ABI-Ambion). 400 ng of each total cellular RNA sample (11 µl) was mixed with an oligo-dT T7 primer (1 µl), 10× reaction buffer (2 µl), dNTP mix (4 µl), Rnase Inhibitor (1 µl), and Arrayscript RT enzyme (1 µl). Reverse transcription was carried out for 2 hours at 42°C. After a 3 minute incubation on ice, the cDNA underwent second strand synthesis by adding water (63 µl), 10× second strand buffer (10 µl), dNTP mix (4 µl), DNA polymerase (2 µl) and Rnase H (1 µl). This cocktail was incubated at 16°C for 2 hours. Following a column cleanup using DNA columns provided in the MessageAmp TotalPrep kit, in vitro transcription was carried out by adding 10× T7 reaction buffer (2.5 µl), T7 biotin-NTP mix (2.5 µl), and T7 RNA polymerase enzyme mix (2.5 µl) and incubated at 37°C. The IVT reaction was carried out for 14 hours. Following reaction termination with water (75 µl), the amplified RNAs [21] were cleaned with RNA columns provided in the MessageAmp TotalPrep kit. The aRNAs were quantitated on a spectrophotometer and quality determined by Agilent 2100 bioanalyzer (Agilent Technologies) according to manufacturer's recommendations.

### Microarray Hybridization, Detection and Analysis

750 µg of each aRNA in water (5 µl) was suspended in Illumina “HYB” buffer (10 µl) and heated to 65°C for five minutes, then allowed to cool to room temperature. The samples were applied to RatRef-12 Expression BeadChips and hybridized at 58°C for 16–20 hours at high humidity. Arrays were washed according to Illumina standard protocol. Immobilized, biotinylated aRNAs were then detected by staining with cy3 streptavidin (1 µg cy3-SA per 1 ml of Illumina “Block E1”) for 10 minutes at room temperature. Arrays were washed and dried according to Illumina standard protocol, then scanned on an Illumina BeadArray Reader. Laser power and PMT voltage were kept constant for cy3 scans. After image quantitation (Illumina Beadscan, v3) data were imported into Beadstudio software. On-slide spot replicates were averaged by Beadstudio and individual spot data were reported. The microarray data discussed in this publication have been deposited in NCBI's Gene Expression Omnibus [22] and are accessible through GEO Series accession number GSE27065 (http://www.ncbi.nlm.nih.gov/geo/query/acc.cgi?acc=GSE27065).

### Microarray Analysis Using Partek® Genomics Suite™

Quantile normalized microarray data were retrieved from the Washington University Genome Technology Access Center and imported into Partek® Genomics Suite™ (Partek Incorporated, 6.4). First, data were filtered to include only data points that had a detection p-value less than 0.01 in all microarrays. This excluded data points that were not significantly different from the background of the chip. Next, the average signal data were log transformed. Principal component analysis revealed one outlier in the day 3 lamellar group. This sample was excluded from all further analysis. Using the Partek “gene expression workflow” to detect differentially expressed genes, an ANOVA was performed. ANOVA factors included sentrix position, chip number, treatment, timepoint, and all contrasts between treatment and timepoint. Gene expression differences between the two loading conditions and between each loading condition and normal were determined using a false discovery rate (FDR) of 0.05 in a step up analysis. From this analysis, gene lists were created comparing each treatment group at each timepoint for a total of nine comparisons (e.g., lamellar day 1 vs. normal; woven day 3 vs. lamellar day 3; Table 2). Exported lists included significant genes, fold changes and p-values for comparisons between groups and timepoints. These lists were then imported into GeneGo® for further analysis.

**Table 2:** Comparisons (nine total) of the number of DEGs between groups at 1 hr, day 1 and day 3; the woven vs. lamellar DEGs were further analyzed using GeneGo software.

	1 hr	Day 1	Day 3
Woven vs. Lamellar	395	5883	5974
Woven vs. Normal	749	5869	4916
Lamellar vs. Normal	5	0	0

### Data Mining Using GeneGo®

Data lists were uploaded into GeneGo® (version 5.4) by accession number. Two separate GeneGo Enrichment Analysis (EA) procedures were performed on the gene lists. GeneGo defines an EA procedure as mapping gene IDs from the dataset onto gene IDs in entities of built-in functional ontologies (represented by canonical pathway maps, cellular process networks, disease biomarker networks, drug target networks, toxicity networks, and metabolic networks). Within each analysis the terms are statistically ranked based on their relevance within the dataset. First, a workflow for each comparison was generated using GeneGo's “Analyze Single Experiment” feature with thresholds of 0.99 and 0.0001 for p-value and fold change, respectively. Non-stringent filters were used in this step as all non-significant expression changes had been excluded by Partek analysis. This workflow resulted in the top ten “Statistically Significant Maps”. Second, a functional EA procedure was done with “GeneGo map folders” for each comparison. This analysis gives a p-value distribution of GeneGo pathway maps folders for each dataset. The EA procedures are limited to canonical pathways represented in the GeneGo database. To overcome this limitation GeneGo has the option to create and study networks generated from user input, which was how most of our data mining was accomplished. Significant pathway maps from the workflow and map folder analysis, along with other canonical GeneGo Pathway Maps were investigated. Relevant pathways reported in the surveyed literature were manually identified and microarray data was examined to select genes of interest. For this more focused analysis, there were several pathways/processes we had selected a priori, including development and osteogenesis, angiogenesis and hypoxia, and bone remodeling. Based on the GeneGo EA results, we also focused on inflammation. The genes listed in Table S3 are only ones that have been referenced within the body of the manuscript and are a subset of all genes differentially regulated in the microarray. Some of these selected genes came to our attention from GeneGo EA analysis while others were selected from literature.

### Quantitative Real-Time PCR

Following microarray analysis, quantitative real-time PCR (qRT-PCR) was performed using the same RNA samples in order to validate expression patterns for select genes, including interleukin-6 (*Il6*; a pro-inflammatory cytokine), nuclear factor of kappa light polypeptide gene enhancer in B-cells 1 (*Nfkb1*; a transcription factor), nuclear factor of kappa light polypeptide gene enhancer in B-cells inhibitor, alpha (*Nfkbia*; an NF-κb inhibitor), toll-like receptor 2 (*Tlr2*; a cell surface receptor implicated in mechanotransduction and inflammation), selectin, endothelial cell (*Sele*; a cell adhesion molecule), prostaglandin-endoperoxide synthase 2 (*Ptgs2*, also known as *Cox-2*; a possible indicator of vasodilation and/or mechanotransduction), chemokine (C-X-C motif) ligand 10 (*Cxcl10*; an angiostatic factor), sclerostin (*Sost*; an inhibitor of the Wnt pathway), matrix metalloproteinase 13 (*Mmp13*; a proteinase capable of cleaving collagen), and cathepsin K (*Ctsk*; a protease involved in bone remodeling). First strand cDNA was synthesized (Superscript III, Invitrogen) from total RNA (500 ng). qRT-PCR reactions were carried out at 20 µl total volume and measured with *Power* SYBR® green (7300 Real-Time PCR System, Applied Biosystems). All primers were purchased as pre-validated sets from Qiagen (QuantiTect Primer Assays; Table 3). Samples were run in triplicate and the average was used for further analysis. Data were analyzed using relative quantification (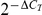), where gene CT values were normalized to glyceraldehyde-3-phosphate dehydrogenase (*Gapdh*).

**Table 3:** Relative fold changes (loaded over normal) for gene expression analysis done using qRT-PCR.

Gene Name	Gene Symbol	Qiagen primer num.	Lamellar			Woven		
			1 hr	Day 1	Day 3	1 hr	Day 1	Day 3
interleukin 6	*Il6*	*QT00182896*	1.2	1.1	−1.2	504.2* #	212.3* #	38.6* #
toll-like receptor 2	*Tlr2*	*QT00417438*	1.1	1.5	1.5	3.9* #	24.2* #	17.8* #
nuclear factor of kappa light polypeptide gene enhancer in B-cells 1	*Nfkb1*	*QT01577975*	−1.0	1.2	1.2	1.6* #	3.3* #	4.6* #
nuclear factor of kappa light polypeptide gene enhancer in B-cells inhibitor, alpha	*Nfkbia*	*QT01600956*	2.2*	1.1	−1.1	2.9* #	1.3	1.8* #
selectin, endothelial cell	*Sele*	*QT00179018*	−1.1	2.0*	1.4	10.4* #	18.5* #	9.6* #
prostaglandin-endoperoxide synthase 2	*Ptgs2 (Cox2)*	*QT00192934*	1.1	1.9*	1.4	16.4* #	16.9* #	8.6* #
chemokine (C-X-C motif) ligand 10	*Cxcl10*	*QT01082354*	1.0	2.3‡	1.5	10.4* #	12.3* #	45.7* #
sclerosteosis	*Sost*	*QT00418558*	−1.3	−1.6	1.4	−3.4* #	−19.8* #	−8.1* #
matrix metalloproteinase 13	*Mmp13*	*QT00385686*	−1.4	−2.7*	−2.6*	1.1	9.2* #	4.3* #
cathepsin K	*Ctsk*	*QT00375599*	−1.1	−1.1	−1.4	−1.1	−1.1	2.9* #
nerve growth factor	*Ngf*	*QT01800344*	0.9	1.3	1.2	3.2* #	22.6* #	14.1* #

*p<0.05 vs normal;

‡ p<0.10 vs normal;

# p<0.05 vs lamellar.

## Results

### Analysis of Differentially Expressed Genes

**Figure 3:**
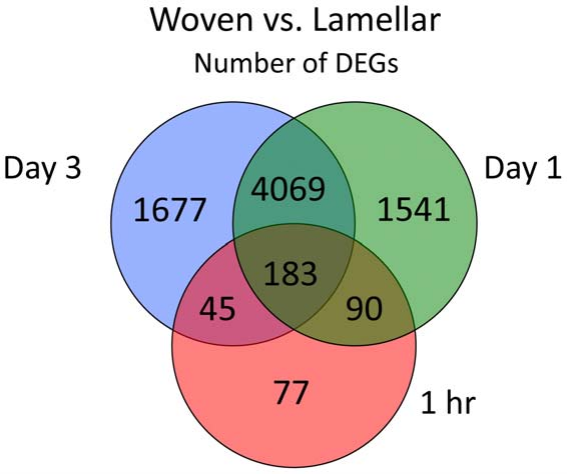
A Venn diagram depicts the commonality of differentially expressed genes. Genes were differentially regulated at 1 hr, 1 and 3 days post-loading for WBF loading (woven) vs. LBF loading (lamellar) groups from microarray analysis.

The number of differentially expressed genes (DEGs) varied when comparing woven, lamellar and normal groups at each timepoint (Table 2). Only five genes (all at 1 hr) were significantly different between normal vs. lamellar groups, whereas thousands of genes were different between woven vs. normal and woven vs. lamellar. Lamellar bone formation is occurring in all samples analyzed, as this is the normal bone formation process in the rat ulna at this young-adult age. The LBF protocol significantly increases the rate of lamellar bone formation without activating woven bone. By contrast, the WBF protocol activates woven bone and increased lamellar bone formation in the region of interest. We chose to focus on woven vs. lamellar comparisons because of the apparent similarity between expression levels in lamellar and normal groups, and because our main objective was to determine the differences between woven and lamellar bone formation. Genes differentially regulated between WBF and LBF loading reflect the woven bone formation process. A Venn analysis of woven vs. lamellar DEGs revealed the commonality between timepoints (Figure 3). A subset of genes (183 genes) was differentially regulated at all timepoints. The vast majority (4069 genes) were common between days 1 and 3. A small number of genes were common between the early timepoint (1 hr) and later timepoints of 1 and 3 days (90 and 45 genes, respectively).

### Top 10 Canonical Pathways

**Figure 4:**
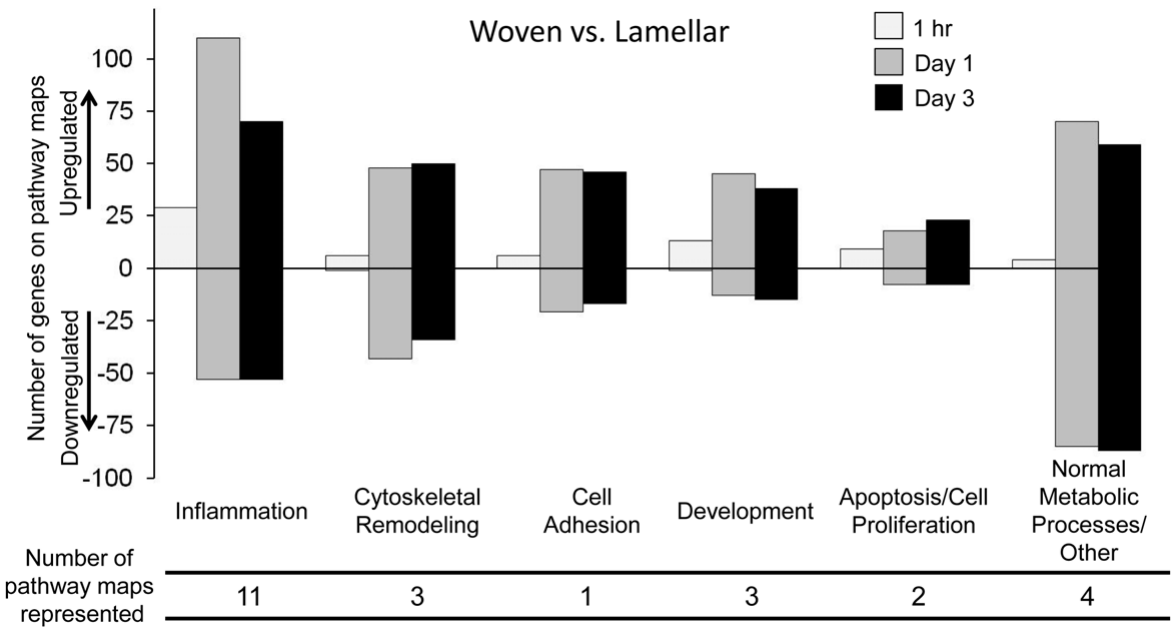
The total number of differentially regulated genes on the top 10 canonical pathway maps. GeneGo enrichment analysis was completed for woven vs. lamellar comparisons at each of the three timepoints investigated. The analysis resulted in a top 10 list of statistically significant canonical pathway maps associated with our data. On each pathway, genes were counted if their expression was significantly different between woven and lamellar bone formation. The number of DEGs on each map (30 maps total) were counted and summed at each timepoint for both upregulated and downregulated genes separately. A total of six pathways were represented at multiple timepoints, although the data were only counted once.

An enrichment analysis done using the GeneGo software calculated the top ten canonical pathways activated by woven vs. lamellar bone formation at each timepoint. At 1 hr after loading the majority of the pathways were related to an inflammatory response (7/10 pathways), plus two pathways related to development. The inflammatory response persisted at day 1 (5/10 pathways) with the addition of cytoskeletal remodeling activation (2/10 pathways). By day 3, the inflammatory response had subsided (0/10 pathways), but cytoskeletal remodeling still played a major role (3/10 pathways) and a development pathway was evident again (1/10 pathways) (Figure 4).

### Map Folder Analysis

**Figure 5:**
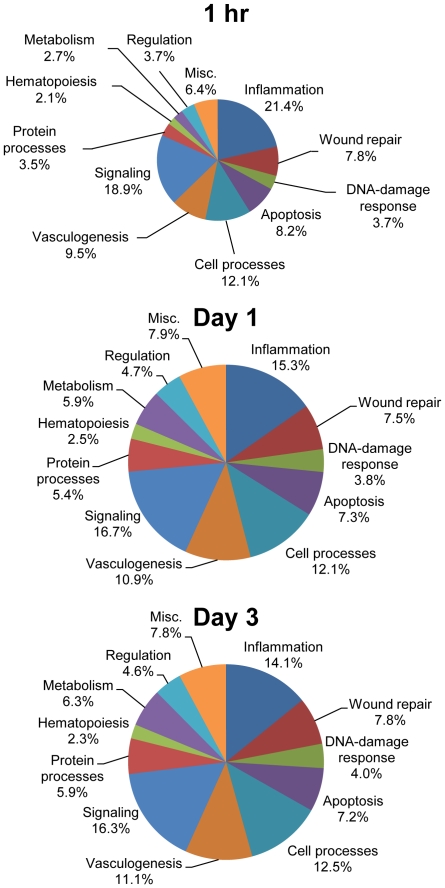
Percentage distribution of statistically significant pathway map folders comparing woven to lamellar bone formation at (A) 1 hr, (B) day 1 and (C) day 3. GeneGo software was used to identify the distribution of the data as mapped onto major functional categories of human metabolism and cell signaling at each timepoint. Out of the 37 available GeneGo pathway map folders, 25 were statistically significant (p<0.05) for at least one timepoint (Table S1). The statistically significant pathway map folders were grouped by the authors for before being displayed in the pie chart. The smaller size of the pie chart at 1 hr reflects the lower number of differentially expressed genes. Within the GeneGo pathway map folders there were 486 differentially expressed genes at 1 hr, 3699 genes at day 1 and 3046 genes at day 3. A complete listing of activated maps is available in Table S2.

In addition to looking at the individual top canonical pathways activated, the distribution of the datasets was examined using GeneGo map folders analysis. The pathway maps as defined by GeneGo are summaries of established, non-contradictory state-of-the-art knowledge on the major functional categories of human metabolism and cell signaling. Maps are assembled into map folders divided onto regulatory, metabolic, disease, toxicity and drug action sections. Differentially expressed genes comparing woven vs. lamellar bone formation were overlaid onto pathway maps and categorized by GeneGo into statistically significant map folders. The map folders were then grouped by the authors in order to better see the distribution of genes associated with known pathways at each timepoint (Figure 5). A breakdown of the GeneGo map folders and the associated number of genes is available in Tables S1 and S2. The total number of genes in each pathway folder varies and some genes are categorized into several folders. Consistent with the top 10 canonical pathway analysis, inflammation was very significant. At each timepoint the most significant pathway related to inflammation.

### Inflammatory Response

WBF loading differentially regulated components of various innate inflammatory response pathways compared to LBF loading (Table S3). One hour after loading several cell surface proteins/receptors know to function in mechanotransduction [23] were upregulated, such as integrins, toll-like receptors (TLRs) and interleukin receptors (specifically *Il1r1*). Toll-like receptor 2 (*Tlr2*) was selected as an indicator of the inflammatory response, and its expression was confirmed by qRT-PCR (Figure 4; Table 3). Both microarray and qRT-PCR data showed *Tlr2* to be upregulated at all timepoints after WBF loading, peaking on day 1 (10- and 24-fold, respectively). *Tlr2* was not differentially regulated for lamellar bone compared to normal at any timepoint by microarray or qRT-PCR. Select intermediate proteins in inflammatory response pathways (such as *Myd88* of TLR pathways) were also upregulated, as were some terminal transcription factors which are responsible for regulating various pro-inflammatory responses, such as NF-κB, AP-1 and NF-AT family members [24] (Table S3).

**Figure 6:**
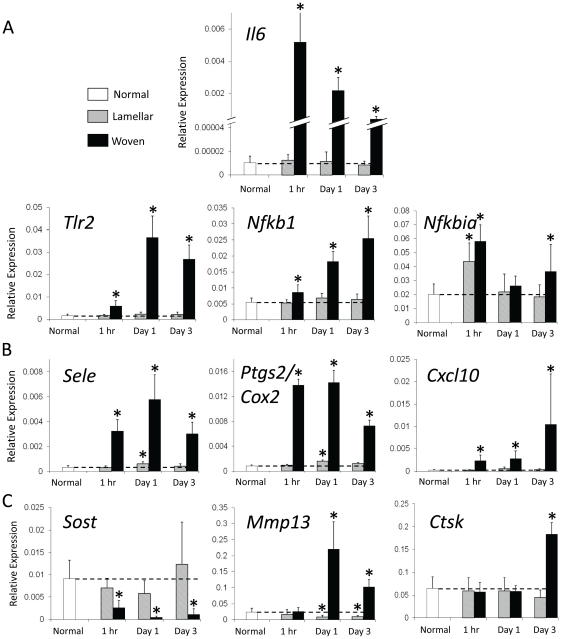
qRT-PCR relative expression (delta CT) of genes related to (A) inflammation, (B) angiogenesis and (C) osteogenesis and matrix remodeling. Inflammatory genes were upregulated at all timepoints for woven bone with the exception of *Nfkbia* on day 1. In contrast, only *Nfkbia* at 1 hr was upregulated for lamellar bone. The inflammation marker *Il6* is increased over 500-fold 1 hr after damaging loading. Both angiogenic markers *Sele* and *Ptgs2/Cox2* are positively related to increased vasculature with expression peaking on day 1 for woven and lamellar bone. In contrast, *Cxcl10* is angiostatic and peaks on day 3 for woven bone but is not significant at any timepoint for lamellar bone. *Sost*, a bone formation inhibitor is downregulated at all timepoints for woven bone. *Mmp13* and *Ctsk* are markers of bone remodeling and both are upregulated at later timepoints for woven bone. In contrast, *Mmp13* is downregulated for lamellar bone. *p<0.05 vs. normal.

NF-κB was of interest as it appeared in a large number of the top 10 significant maps produced by GeneGo from various timepoints. Different combinations of NF-κB subunits form active dimers and several of these subunits (*Nfkb1*, *Nfkb2* and *Rel*) were upregulated in woven as compared to lamellar groups (between 1.5- and 2.5-fold). *Nfkb1* was chosen for confirmation by qRT-PCR and was upregulated at all timepoints for woven bone compared to lamellar bone and normal controls (Figure 6, Table 3). Lamellar expression did not differ from normal controls at any timepoint for *Nfkb1*. Other components of the NF-κB signaling pathway were also differentially regulated for woven bone, including members of the I-κB family such as *Bcl3*, *Nkbiz* and *Nkbie*. *Nfkbia*, which is both an inhibitor and a transcriptional target of NF-κB [25], was upregulated. In addition, *Nfkbia* was one of the five genes to be differentially regulated in the microarray lamellar vs. normal comparison at 1 hr (2-fold upregulation), and this was confirmed with qRT-PCR.

The microarray also showed the upregulation of transcription factors, as well as several targets of NF-κB including pro-inflammatory cytokines (*Il1a*, *Il1b*, *Il6*) [26], chemokines (*Cxcl1*, *2* and *10*) and cell adhesion molecules (*Icam1*, *Sele*). Interleukin 6 (*Il6*) and both the alpha and beta subunits of *Il1* were significantly upregulated (more than 18-fold). Several receptors (*Il1r1* and *Il8rb* for example) were also upregulated. *Socs1*, which takes part in the negative feedback of cytokines [27], was also greatly upregulated across all three days (ranging between 6- and 13-fold) suggesting that mechanisms for the attenuation of the inflammatory response were also active in this time range. Interleukin-6 was chosen as an indicator of pro-inflammatory cytokines. In our study, qRT-PCR confirmed the dramatic upregulation of *Il6* seen for woven bone in the microarray. Expression of *Il6* was upregulated at 1 hr by over 500-fold on qRT-PCR, and near 50-fold on the microarray (Table 3). Like in the microarray, expression for woven bone peaked at 1 hr and declined thereafter with levels still well above normal. Expression for *Il6* was not significant for lamellar compared to normal on the microarray or by qRT-PCR.

### Angiogenic Response

Angiogenesis has been shown to increase in response to WBF loading [5]–[7] and the differential regulation of a variety of angiogenic genes in the microarray supports this. Several factors related to HIF-1a, a central component of angiogenesis-osteogenesis coupling [28], were differentially regulated in woven bone. The constitutive beta subunit (*Arnt*) of HIF-1a was upregulated (1.7-fold), as were the inducible alpha subunit and coactivators found in the nucleus such as *Creb1* (near 2-fold) after WBF loading. Moreover, a HIF-1a inhibitor, *Egln1*, was downregulated (2-fold).

Chemokines, in addition to their role in inflammation, play a role in angiogenesis [29], [30]. In our study, *Cxcl1*, *-2*, *-6* and the receptor *Il8rb* were upregulated (between 8- and 66-fold) while *Ppbp* was downregulated (near 8-fold) for woven bone formation. Moreover, WBF loading upregulated angiostatic chemokines [29], [30], including *Cxcl10* and *-13* (3- to 12-fold), and the CXCL-10 receptor *Cxcr3* (1.8-fold). *Cxcl10* was selected for further confirmation by qRT-PCR and was significantly upregulated 1 hr (10-fold) after WBF loading and continued to increase through day 3 (46-fold) (Figure 6, Table 3). WBF loading upregulated another chemokine and its receptor, *Ccl20* and *Ccr2* respectively, (greater than 85-fold for *Ccl20*), along with other genes influencing chemotaxis such as *Ccl7* (6- to 55-fold) [31]. *Sele* was chosen to represent chemotaxis in the endothelia [32] and for further confirmation by qRT-PCR. The microarray showed *Sele* to be upregulated at all three timepoints for woven bone (2- to 7-fold), peaking at 1 hr. The qRT-PCR showed *Sele* to be upregulated significantly in the woven group, but in contrast to the microarray, expression peaked on day 1 at 19-fold (Figure 6, Table 3). Expression of *Sele* was upregulated at day 1 (2-fold) in the lamellar group compared to normal by qRT-PCR.

Genes associated with blood flow regulation and vasodilation were also differentially expressed. Following WBF loading *Ptgs2*/*Cox2* was upregulated in the microarray data (2- to 9-fold), as was *Nos3* (a constitutive form of NOS), and *Creb1* (a transcription factor for *Ptgs2*/*Cox2*). *Cebpb*, a regulator of COX-2, was upregulated at 1 hr and day 1 (1.6-fold) but downregulated on day 3 (1.5-fold). *Ptgs2/Cox2* was selected for verification by qRT-PCR and was upregulated at all timepoints in the woven group (9- to 17-fold), but declined towards normal by day 3 (Figure 6, Table 3). Following LBF loading, *Ptgs2/Cox2 was* upregulated on day 1 (1.9-fold) by qRT-PCR.

### Osteogenic Response

Mechanical loading is known to greatly influence bone modeling [33], [34], and various aspects of this process were differentially regulated for woven bone in our microarray, including possible mechanoreceptors such as cadherins (for example *Cdh16*, *Fat3* and *Cdh19*) and integrins (*Itgax*, *Itgal*, *Itgam* and *Itgb2*) (Table S3). Though several DEGs were part of the canonical Wnt-pathway, the pathway as a whole was not clearly or dramatically regulated in one direction. Wnts were not differentially regulated though their receptors were. *Lrp5* was suppressed as was *Fzd8* (1.4- to 1.8-fold), while *Fzd1* was slightly upregulated (1.6- to 1.7-fold). *Dkk1* and *Sfrp4* (inhibitors) were downregulated (1.5- to 6-fold). Both Dishevelled (*Dvl1*) and casein kinase I (*Csnk1a1*) form a complex with beta-catenin, targeting it for ubiquination (i.e. suppressing the pathway). Though *Dvl1* was downregulated (2-fold), an isoform of *Csnk1a1* was upregulated (1.5-fold) as was one of its nuclear partners, *Tcf7* (2- to 4-fold). While the direction of regulation of the pathway in general was rather ambiguous, sclerostin (*Sost*) was clearly affected. Consistent with previous studies [35], [36], *Sost* was greatly suppressed in the microarray after WBF loading (16- to 18-fold). qRT-PCR of *Sost* for woven bone confirmed this pattern (downregulated 20-fold on day 1) (Figure 6). *Sost* was not differentially regulated comparing lamellar bone to normal in either the microarray or by qRT-PCR.

BMP pathways were also differentially regulated in woven as compared to lamellar bone formation. TGF-β may initiate BMP synthesis, as well as having chemotactic, proliferative and matrix remodeling effects of its own [26]. Our data show mixed regulation of three types of TGF-β for woven bone: *Tgfb1* was upregulated (2-fold), *Tgfb2* was downregulated (2-fold), and *Tgfb3* was downregulated on day 1 (1.7-fold) and upregulated on day 3 (1.5-fold). *Bmp2* itself was upregulated, though only slightly, while *Bmp3*, -*3b (Gdf10)*, - 4, -6 and -7 were downregulated (1.6- to 4-fold). The receptors for BMP-5, 6, 7, and 8, *Acvr2b* and *Acvr1* were also downregulated (1.3- to 2-fold) for woven bone. The downstream transcription factors *Smad1* and *Smad5* were slightly upregulated (1.3- to 1.7-fold). The BMP pathways were most clearly modulated by the downregulation of antagonists [37] such as the previously mentioned *Sost*, as well as *Nbl1*, *Bambi*, *Fst*, *Grem1*, *Chrd* and inhibitory SMADs (*Smad6* and *Smad7*) (1.7- to 18-fold) (Table S3). No changes in BMP pathway regulation were seen when comparing lamellar bone to normal controls.

### Bone Remodeling and Cell Proliferation Response

Genes associated with matrix remodeling, specifically those expressed by osteoclasts, were also differentially regulated for woven bone. Members of the cathepsin family (*Ctsd*, *Ctsb*, *Ctss* and *Ctsl1*) [38] along with *Csf1* and *Myc* were upregulated to some extent (1.6- to 5-fold). Cathepsin K (*Ctsk*) localizes predominantly in osteoclasts [39], [40] and was chosen as an indicator of osteoclast activity for confirmation with qRT-PCR (Figure 6, Table 3). *Ctsk* was slightly downregulated for woven bone on day 1 in the microarray (1.5-fold), whereas qRT-PCR showed it to be at normal levels at 1 hr and day 1 but upregulated on day 3 (3-fold). There were no *Ctsk* differences on qRT-PCR for lamellar bone compared to normal controls. Neither RANK nor its receptor was differentially regulated for either woven or lamellar bone formation at any timepoint.

Matrix degradation is an important feature of remodeling. MMPs in general function in this capacity [40], [41] and *Mmp13* was chosen as an indicator of these processes. In the microarray, *Mmp13* was upregulated greatly for woven bone on day 1 (21-fold), and less so on day 3 (4-fold), a pattern seen again in qRT-PCR (Figure 6). Important transcriptional regulators of MMP-13 were also differentially regulated for woven bone: some subunits of AP-1 were slightly up and others slightly down, while *Etv4* was much more strongly downregulated (4- to 13-fold).

Many genes related to cell proliferation were differentially regulated in the microarray for woven bone. Cell cycle regulation genes such as *Ccnd1*, *Cdk4*, *Cdc2*, and *E2f1* were upregulated (1.4- to 6-fold), while *E2f6* and members of the Rb family (*Rbl2*, *Rbl1*) were downregulated (1.3- to 3-fold).

## Discussion

The objective of our study was to identify gene expression differences between woven and lamellar bone formation induced in the adult skeleton using two well-characterized mechanical loading protocols. Periosteal woven bone forms at the midshaft of the ulna in response to WBF loading, whereas lamellar bone forms in response to LBF loading [5]. Comparisons between WBF and LBF loading reflect the woven bone formation process. Using a microarray we saw a dramatically higher number of DEGs for woven compared to lamellar bone formation. Nearly 400 genes were differentially expressed between woven and lamellar bone formation 1 hr after loading, and nearly 6000 genes were differentially expressed on days 1 and 3. There were many inflammatory factors upregulated as early as 1 hr after WBF loading, such as toll-like receptors and pro-inflammatory cytokines. We expanded our previous results related to angiogenesis [5], finding many differentially regulated genes associated with hypoxia and vasodilation. We also examined osteogenesis-related genes, including a detailed analysis of the Wnt and BMP pathways, and found that stimulation of woven bone formation both activates and attenuates various genes along these pathways. Finally, results demonstrated activation of bone remodeling pathways at days 1 and 3 following WBF loading.

An increase in inflammation is seen as the primary response after complete bone fracture [13], [14], [42], and our data, consistent with others [8], [12], indicate a similar response for stress fracture healing. In other studies, expression of the inflammatory factor IL-6 was highly upregulated after stress fracture formation (woven bone response) [8] but not after mechanical stimulation of lamellar bone formation [12]. Our results confirm that *Il6* is highly upregulated in woven, but not lamellar bone. Many cytokines, including *Ccl7* and *Cxcl13*, were upregulated in our study of woven bone and confirm previously published results [12]. Consistent with our single-bout loading results, inflammatory pathways were less up-regulated at later timepoints following both multiple-bouts of loading [12] or a single bout of loading [8] in other studies.

Most of the canonical pathways represented by the GeneGo maps focused on innate inflammatory responses and were in general upregulated. These pathways lead to transcription factors such as NF-κB, which regulates numerous pro-inflammatory responses including cytokines (Il1, Il6, TNF-alpha), chemokines (IL-8) and cell adhesion molecules (ICAM1, E-selectin). In general, a wide range of pro-inflammatory genes known to be targets of these pathways were activated in our data set, specifically cytokines. Proinflammatory cytokines such as IL-1, IL-6 and TNF-alpha have been shown to be secreted by macrophages and mesenchymal cells located in the periosteum after fracture, with expression peaking at day 1 [43]. While TNF-alpha was not differentially regulated in the microarray, *Il6* and both *Il1a* and *Il1b* were greatly upregulated 1 hr after loading, with expression values peaking between 18- and 53-fold, and declining toward normal by day 3 (2- to 5-fold). IL-6 and other pro-inflammatory cytokines serve as a central hub for other down-stream responses to tissue damage, including angiogenesis, ECM synthesis and chemotactic effects on other inflammatory cells [33]. Evidence of these pathways and more were seen in the microarray data. The overall regulation of NF-κB signaling during woven bone formation is complicated by the numerous autoregulatory feedback loops that attenuate the NF-κB response. Attenuation of the immune response was also occurring, as evidenced by increased *Socs1* expression. The inflammatory response during the early stages of woven bone formation requires further investigation.

Prior studies have reported an increase in vasculature [6] and in individual genes associated with angiogenesis [5], [7], [8] following woven, but not lamellar bone formation. In the microarray, genes both promoting and attenuating the angiogenic response were upregulated for woven bone. VEGF-A is considered crucial for angiogenesis and it was upregulated at all three timepoints in previous studies [5], [7], although the microarray showed no significant differential regulation of VEGF-A, or of its receptors. HIF signaling is central to angiogenesis-osteogenesis coupling in osteoblasts, particularly because of its regulation of VEGF [44], [45]. Interestingly, recent studies have shown that Vegf can be induced independently of Hif1a, by Ppargc1a [46], which was greatly downregulated for woven bone in the microarray. VEGF transcription is also promoted under hypoxia conditions by K-ras, as well as JunB and NF-κB. All three were upregulated for woven bone to some extent (1.5- to 6-fold). These findings suggest a more complex network of VEGF regulation that could be further investigated. Additionally, microarray findings also pointed to a number of anti-angiogenic genes that were activated. CXCL-10 in particular, it is thought to be involved in (or perhaps triggers) the involution of microvasculature, when angiogenesis stops or even regresses as the nutrient demand of the tissue decreases. Our results demonstrate a significant upregulation of *Cxcl10*, with expression peaking on day 3 for woven bone. This suggests that *Cxcl10* may be a key negative regulator of the angiogenic response. This and other anti-angiogenic factors brought to light by the microarray could be further examined during woven bone formation.

Also related to a vascular response, the microarray data indicated upregulation of prostaglandin signaling and vasodilation factors, such as *Ptgs2/Cox2*. This phenomenon has seen by others in fracture healing [47], stress fracture healing [8], and mouse tibial loading [19]. Following LBF loading there was a significant increase in *Cox2* expression only at day 1 (2-fold). There have been reports that *Cox2* expression is *not* required for lamellar bone formation in the ulna [48], [49]. Vasodilation is regulated to some extent by the biosynthesis and release of nitric oxide and prostaglandins, controlled by constitutive and inducible forms of nitric oxide synthase (NOS) and cyclooxygenase, respectively [50], [51]. The inducible isoforms are sensitive to inflammation, and osteocytes activated by fluid shear stress have been also shown to produce both prostaglandins and nitric oxide (NO) [33], both of which were highly upregulated for woven bone in our study. In contrast to our microarray results, which did not suggest a relationship between NO and lamellar bone formation, inhibition of NO was shown to decrease endocortical lamellar bone formation in the rat tibia [52]. These differences may be due to sensitivity of the microarray or to the skeletal site studied, as *Cox2* inhibition experiments in the rat have shown differences in lamellar bone formation between the tibia and ulna [49].

Mechanical stimulation, such as the loading done in this experiment, greatly influences bone formation [2], [4]. Similar to another microarray study on mouse tibiae [11], a number of bone-related genes were upregulated in our study. The canonical Wnt signaling pathway is central to bone remodeling and has been shown to be directly affected by mechanical loading through the suppression of sclerostin (*Sost*) in osteocytes [33]. *Sost* normally inhibits the Wnt-pathway, in particular through its interactions with LRP5 [23]. Therefore, a suppression of *Sost* would lead to an increase in osteogenesis. In our study, *Sost* expression was highly downregulated after WBF loading, consistent with the large amount of woven bone formed 7 days after loading [2], [5]. Pathways involving BMPs, specifically BMP-2 are critical for osteogenesis. TGF-β may initiate BMP synthesis, as well as having chemotactic, proliferative and matrix remodeling effects of its own [26]. Although we have seen increased expression in *Bmp2* for both woven and lamellar bone in past qRT-PCR experiments [5], [7], expression of *Bmp2* in the microarray was only evident for woven bone formation on day 1. In contrast, other BMP's in the microarray were downregulated at various timepoints. In agreement with our findings on BMPs, a microarray fracture study also found weak or no expression of common bone morphogens including BMP2, 4, 6 and 7 [14].

Bone remodeling is an important part of the damage-repair process. The tissue sample used for microarray analysis consisted of both new periosteal bone and original cortical bone. After WBF loading, there are large cracks and visible diffuse damage in the cortex [5]. Repair of the cracks in the cortex of the ulna begins with osteoclast activation [53]. Our upregulation of matrix remodeling genes for woven bone (including *Ctsk* and *Mmp13*) may be due to remodeling at sites of damaged matrix in the cortical bone. Alternatively, upregulation of *Mmp13* could be a result of osteoblast differentiation as increased expression was seen even after mild tibial loading in mice [11]. Promoters of MMP-13 are known to be upregulated by RUNX2 during osteoblast differentiation [54]. Our previous study demonstrated upregulation of *Runx2* during woven, but not lamellar bone formation [5].

One additional area to consider is the nervous system response to loading because of its role in fracture healing [15], [16] and recent work suggesting a possible role in bone formation at non-loaded sites [55]. Additional skeletal sites were not examined in this study, but a recent study demonstrated no evidence of bone remodeling in contra-lateral limbs [56]. Microarray data demonstrated a significant increase in nerve growth factor (NGF) in response to WBF loading, which was confirmed using qRT-PCR (Table 3). The response was upregulated as early as one hour after loading, but only for woven bone formation. Lamellar bone did not show any increase in NGF expression. Both multi-day loading [12] and our single-day loading inhibited various neurotransmitter transporters in the solute carrier family including *Scl4a1*, *Slc6a4* and *Scl25a11*.

One unexpected result from our study was the lack of difference between LBF loading and normal ulnae (non-loaded controls) in the microarray data. Only 5 genes were differentially regulated 1 hr after loading. It is possible that our selected timepoints did not capture the most robust expression timeframe for lamellar bone. Other studies have looked at timepoints including 4, 8, 12 hr after loading while our study looked only at 1 hr, 1 day and 3 days post loading. Our selected timepoints for expression analysis were based on prior studies and coincided with previously large changes in expression following woven bone formation in stress fracture healing. It is also possible that the number of cells responding to LBF loading is small compared to all the cells in the bone-plus-periosteum sample analyzed. Thus, changes in expression of these responsive cells may be diluted by the relatively larger number of non-responsive cells contributing to the RNA pool.

There are some other limitations to our study. First, with nearly 6000 genes differentially expressed at days 1 and 3 for woven bone, we were not able to complete a detailed analysis of most individual genes. To address this limitation we used commercially available software for microarray analysis. Powerful statistical packages, such as Partek Genomics Suite are available to manage the vast amount of data (over 22,000 genes) created from a single microarray. Furthermore, a variety of pathway analysis tools, such as GeneGo, which are constantly edited and updated to reflect current literature are a valuable resource to identify pathways and connections between genes both expected and unanticipated within the microarray results. Second, we limited our initial data mining to the GeneGo workflow, generating statistically significant maps from canonical pathways available in the GeneGo software. This bias was incorporated into our overview analysis which included the top ten canonical pathways and pathway map folder analyses. After examining relevant pathways in our data we created and studied networks generated from user input. Finally, differences in sensitivity between microarray and qRT-PCR were demonstrated in our data. While the fold change differences in expression were not identical, we saw the same general patterns of increasing or decreasing expression using the two methods.

A microarray is an extremely powerful tool that can be used to assess the expression of thousands of genes simultaneously. Using this assay, we were able to identify molecular responses that differed between woven and lamellar bone formation. Continued mining of this data and other publically available data sets will lead to a more complete and comprehensive overview of the molecular signaling events activated by mechanical loading. Our study provides a unique data set including an overview of the response of woven bone simultaneously compared to an *only* lamellar bone formation scenario so that responses unique to woven bone can be examined. A complete understanding of the molecular mechanisms that regulate bone formation in the adult skeleton will be informative for development of drugs or therapies to help promote bone formation in clinical settings.

**Figure 7:**
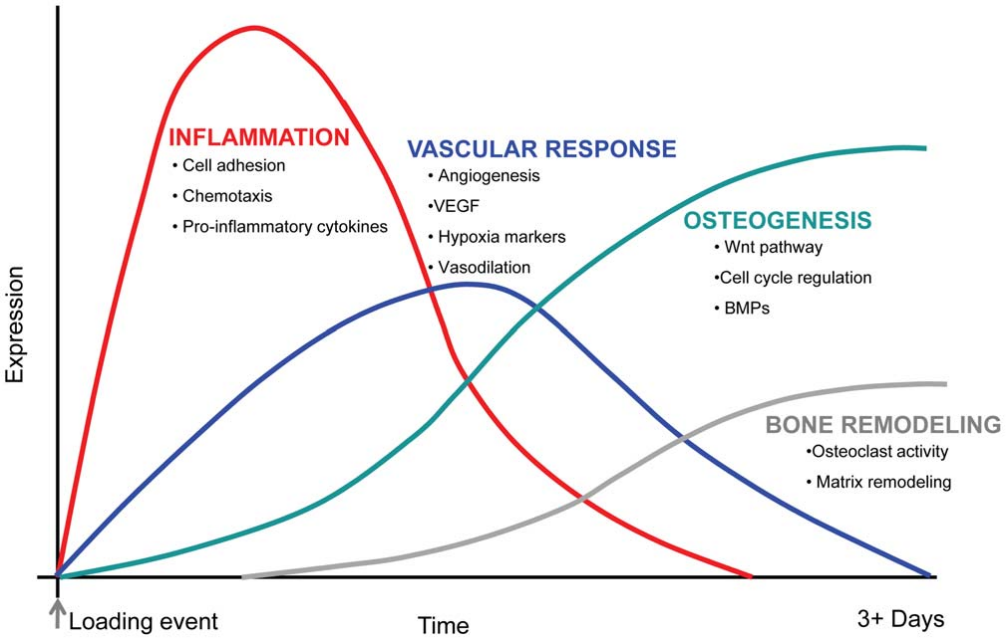
In combination with our previous report [**5**], we have created an overview of the early molecular response comparing woven to lamellar bone formation. There is an early immune response that persists through time but tends to decrease in expression. The vascular response is also a major component of woven bone formation and it precedes osteogenesis. Osteogenic indicators are differentially regulated shortly after loading, but seem to increase over time. Finally, bone remodeling markers are activated later, possibly to repair bone damage. Gene expression changes likely persist through many weeks after loading, but our data only includes early timepoints after loading.

In closing, the vast amount of data present in the microarray can be used to show how expression of genes is choreographed to promote the sequential steps in bone formation. An overview of the major responses associated with woven bone formation depict a strong early inflammatory response, followed by an increase in angiogenesis and upregulation of osteogenic genes, and finally an activation of bone remodeling gene expression (Figure 7). Validation of these pathways using select target genes (qRT-PCR) confirmed their differential expression in our study. Use of a whole genome microarray has allowed us to confirm some gene expression responses from our prior study [5] and has identified other genes and pathways that differ between woven and lamellar bone formation.

## Supporting Information

Table S1 GeneGo map folder analysis of the number of differentially regulated genes (DEGs) at three timepoints (1 hr, day 1 and day 3).
(XLSX)

Table S2 Complete breakdown of individual map folders from GeneGo map folder analysis identifying the number of differentially regulated genes (DEGs) from Table S1.
(XLSX)

Table S3 Summary of genes referenced throughout the text (fold change between woven and lamellar bone formation); the list is a subset of DEGs from the microarray based on a combination of pathway analysis and literature reviews.
(XLSX)
